# The influence of a brittle Cr interlayer on the deformation behavior of thin Cu films on flexible substrates: Experiment and model

**DOI:** 10.1016/j.actamat.2015.01.047

**Published:** 2015-05-01

**Authors:** Vera M. Marx, Florian Toth, Andreas Wiesinger, Julia Berger, Christoph Kirchlechner, Megan J. Cordill, Franz D. Fischer, Franz G. Rammerstorfer, Gerhard Dehm

**Affiliations:** a Max-Planck-Institut für Eisenforschung GmbH, Max-Planck-Str. 1, D-40237 Düsseldorf, Germany; b Department Material Physics, Montanuniversität Leoben, Jahnstr. 12, A-8700 Leoben, Austria; c Institute of Lightweight Design and Structural Biomechanics, Vienna University of Technology, Gußhausstr. 27-29, A-1040 Vienna, Austria; d Erich Schmid Institute for Materials Science, Austrian Academy of Science, Jahnstr. 12, A-8700 Leoben, Austria; e Institute of Mechanics, Montanuniversität Leoben, Franz-Josef-Str. 18, A-8700 Leoben, Austria

**Keywords:** Thin films, Synchrotron diffraction, Tensile test, Finite-element simulation

## Abstract

Thin metal films deposited on polymer substrates are used in flexible electronic devices such as flexible displays or printed memories. They are often fabricated as complicated multilayer structures. Understanding the mechanical behavior of the interface between the metal film and the substrate as well as the process of crack formation under global tension is important for producing reliable devices. In the present work, the deformation behavior of copper films (50–200 nm thick), bonded to polyimide directly or via a 10 nm chromium interlayer, is investigated by experimental analysis and computational simulations. The influence of the various copper film thicknesses and the usage of a brittle interlayer on the crack density as well as on the stress magnitude in the copper after saturation of the cracking process are studied with in situ tensile tests in a synchrotron and under an atomic force microscope. From the computational point of view, the evolution of the crack pattern is modeled as a stochastic process via finite element based cohesive zone simulations. Both, experiments and simulations show that the chromium interlayer dominates the deformation behavior. The interlayer forms cracks that induce a stress concentration in the overlying copper film. This behavior is more pronounced in the 50 nm than in the 200 nm copper films.

## Graphical abstract

**Figure:**
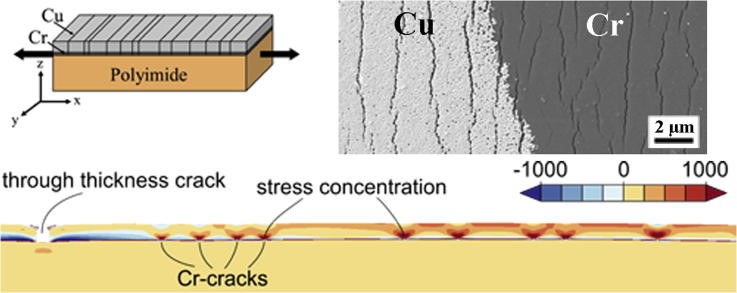

## 1 Introduction

In order to advance the reliability of flexible electronics such as paper-like displays or flexible sensors [1–6], it is important to investigate and understand the mechanisms which lead to mechanical and electrical failure of the whole device. These lightweight and flexible devices have to work under different environmental conditions like bending, compressive, or tensile forces as well as under various temperatures. Delamination of the conducting metal film (usually Cu or Ag) from the substrate may result in the loss of electrical functionality. To improve the adhesion strength different brittle interlayers can be used, for example Cr, Ta or Ti [7–10], which form a strong bond with the C atoms of the polymer [11,12]. Oxygen plasma treatments prior to film deposition have also shown to improve the adhesion of Cr to a polyimide substrate [13].

In this study, the mechanisms leading to a mechanical failure of 50, 100 and 200 nm thick Cu films with and without a 10 nm thick Cr interlayer (in the following called as Cu or Cu/Cr films) on polyimide were investigated experimentally, using tensile tests combined with synchrotron diffraction to observe in situ the film stress evolution under load. Gruber et al. [10] already showed that synchrotron diffraction is a versatile tool to successfully study the mechanical properties of very thin Cu, Cu with a Ta interlayer, and Ta/Cu/Ta films on polyimide with thicknesses between 20 nm and 1 μm. In this case, the Ta layers confined the Cu films either on one or on two sides which lead to an increase of the flow stress compared to bare Cu films. Furthermore, in situ straining with synchrotron diffraction allows the determination of the film stresses parallel as well as perpendicular to the tensile direction. Due to a mismatch of the Poisson’s ratio between the thin films and the substrate leading to transverse contractions, transverse stresses arise in the film [9,10,14]. Moreover, transversal cracks reduce longitudinal strains in their vicinity in the film, according to the formation of a shear lag. This, in combination with strain coupling in the transverse direction, leads to transversal compression of the film, even if no Poisson’s mismatch exists. In this respect, necking of the film acts similar to the above described effect of cracking. Due to the longitudinal unloading in the film caused by cracking or necking a relation between the stress state in the film and development of the crack (or neck) density exists.

In comparison to brittle films which form channel cracks under tensile loading [15,16] and at higher strains buckles (film delamination) perpendicular to the tensile direction [17–19], thin ductile films bonded to polymer substrates form necks (localized thinning) all over the film. Necking is a material instability leading to localized large plastic deformations. Freestanding ductile films commonly fail at much lower global strain levels (i.e. the necking limit) than the bulk material, which can be explained by the formation of necks and, thus, high local strains leading to failure [20]. If films are bonded to a substrate, the localization cannot evolve freely. The substrate has a “delocalization” effect, which allows the films to be strained far beyond the necking limit without failure [20]. In some studies [21,22], an adhesion interlayer retards the strain localization and cracking of thick Cu films due to the prevention of the debonding. However, as will be presented through experiments and simulations, a brittle Cr interlayer can influence the delocalization effect because cracking of the brittle interlayer consequently leads to necking and cracking of the overlying Cu film.

In the literature [19,20,22,23], several finite element simulations of the deformation mechanisms leading to a failure of thin films on flexible substrates under load can be found. However, the simulation of such material systems is challenging: Cracking of the film is commonly treated by cohesive zone models, and macroscopic constitutive laws can be used to describe the material behavior. To get accurate modeling results, precise knowledge of the material properties is important, however, for most material parameters assumptions have to be made, because it is still difficult to determine them experimentally. Bulk parameters cannot be directly applied to thin films, since the plastic properties of crystalline solids are known to be size dependent [24–26], with a transition from a bulk-like (macroscopic) to a stochastic behavior. That transition depends on several microstructural and geometrical parameters. Additionally, the usually applied 2-D modeling cannot successfully describe the complex 3-D deformation of the real devices such as for example the delamination of the interface in the form of buckles. Therefore, Toth et al. [19] performed a 3-D multi-scale finite element simulation to model the deformation behavior of a brittle Cr film on polyimide under tensile load, especially the delamination processes at the interface. This model used a new two-stage simulation applying cohesive zones to describe the full load transfer between the film and the substrate as well as the shear stresses at the interfaces leading to delamination of the film. Another group [22] also implemented cohesive zones in a 2-D plane strain model to simulate the failure mechanisms of Cu films on polyimide under tensile load. In this case, the films fail by a coevolution of film debonding from the substrate and necking, depending on the yield strength of the film. Their simulations showed that the film can suppress the delamination as well as the necking of the film, if the yield strength is too low compared to the interfacial strength. However, their simulations did not take into account the deformation behavior when using an interlayer improving the adhesion.

In the present paper, the experimental results will be explained and compared with 2-D finite element simulations using a generalized plane strain model and cohesive zones to model the evolution of the crack pattern in the Cu/Cr film system under tensile load. A 2-D model is adequate to successfully describe the cracking behavior of the film system. The experiments, using in situ tensile tests combined with synchrotron diffraction and under an atomic force microscope (see Section 2), and the modeling (see Section 3) will show that the brittle Cr interlayer dominates the deformation behavior in Cu/Cr films compared to bare Cu films and acts as a stress concentrator leading to cracking of the overlying Cu film.

## 2 Experimental methods

### 2.1 Mechanical experiments

The studied material system consists of thin Cu films deposited onto a 50 μm thick polyimide (UPILEX) substrate. In order to investigate the influence of an adhesion layer between the Cu film and the substrate on the deformation behavior, one set of samples contains Cu films without an interlayer while a second set contains a 10 nm Cr interlayer between the Cu films and the polyimide. All of the Cu films were fabricated by electron beam evaporation in a Balzers BAK 550 evaporation machine with a deposition rate of 0.5 nm/s and a vacuum of 5 × 10^−7^ mbar to thicknesses of 50, 100, and 200 nm. The Cr interlayer of the second set of samples was deposited in the same manner using the same deposition rate but with a vacuum of 2 × 10^−7^ mbar. For comparison, a bare 10 nm Cr film, deposited the same way as the Cr interlayer, was also investigated. The grain sizes of the Cu and Cu/Cr films were determined by different electron microscopy techniques [14]. The 50 and 100 nm Cu films have an average grain size of 39 ± 14 nm and 77 ± 30 nm, respectively. The Cu/Cr films have a similar average grain size of 36 ± 12 nm and 65 ± 21 nm, respectively for the 50 and 100 nm Cu/Cr films. The average grain size was evaluated by measuring the grain diameter in TEM images of over 30 grains for each film system. In contrast, the grain sizes of the 200 nm Cu and Cu/Cr films were large enough to use electron backscatter diffraction (EBSD) in a JEOL 6500 scanning electron microscope (SEM) which also provides information about the grain orientation. Again, both Cu films have a similar bimodal microstructure. The EBSD scans of the Cu/Cr film show small grains with a size of about 100 nm with a predominant [1 1 1] direction out of plane and larger 1–2 μm grains with predominant [1 0 0] orientation embedded by these smaller grains. In comparison, the 200 nm Cu film was not strongly textured like the Cu/Cr film. The bare Cr film has an average grain size of 2.5 ± 0.7 nm determined from TEM images analyzing 50 grains.

**Fig. 1:**
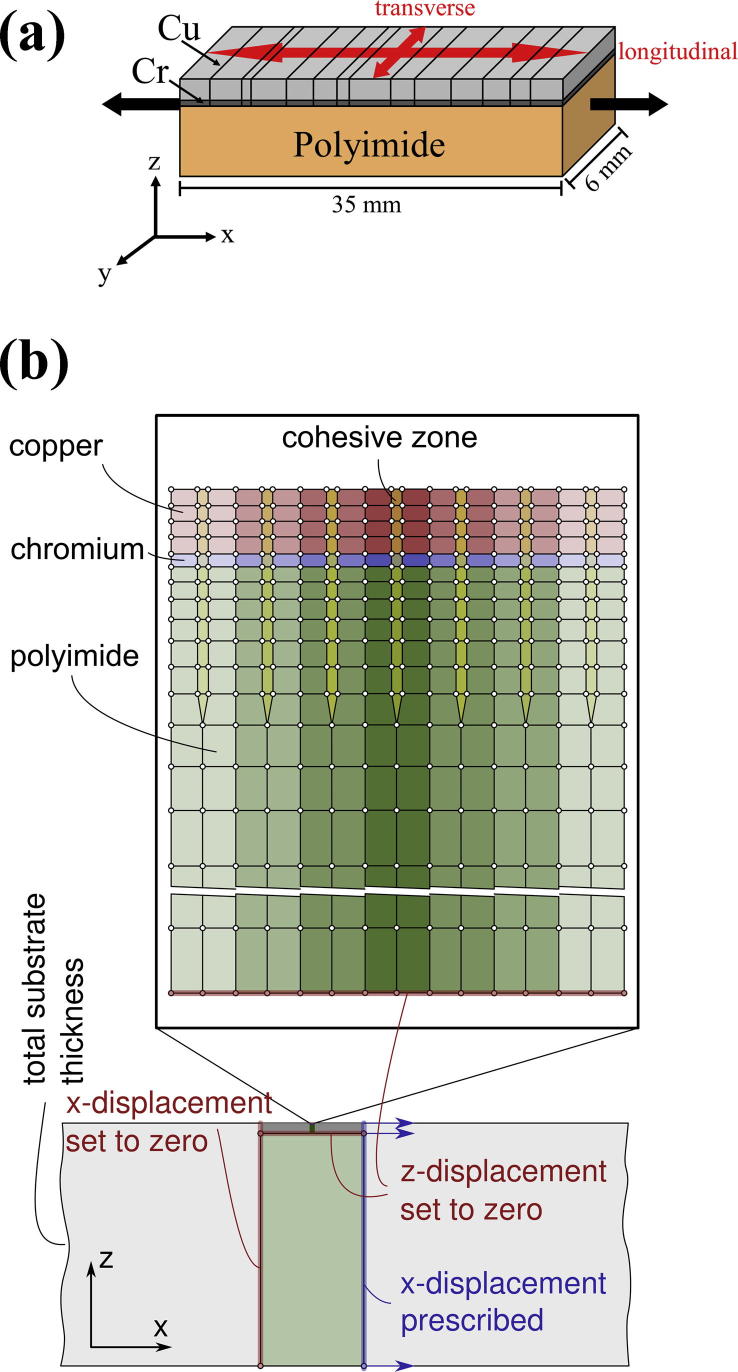
(a) Schematic diagram of the straining samples with the longitudinal and transverse directions indicated. (b) Sketch of the FE model in generalized plane strain configuration. The figure on top shows a zoom-in of a very small detail of the whole FE-model, shown below.

In situ diffraction tensile tests were performed in the synchrotron BESSY II in Berlin (Germany) at the beam line KMC-2 [27] to link the observed deformation behavior with the stresses of the Cu films under load. Therefore, the samples, cut to the size of 6 mm × 35 mm using a scalpel (Fig. 1a), were strained continuously to a maximum engineering strain of 12% and then unloaded to 0 N using a commercial Anton Paar TS 600® straining device which recorded the load, displacement, and time of the whole sample (film and substrate) during the experiment. Simultaneously, the (1 1 1) Cu peaks, using six or eleven different *ψ* angles, were measured prior to the tension test for evaluation of the residual stresses. In situ measurements at four different *ψ* angles were performed with a Bruker VÅNTEC detector and an exposure time of 5 s for all 200 nm films and 10 s for all 50 nm and 100 nm films. In the case of the 200 nm Cu/Cr film, the (2 0 0) Cu peak was chosen in the longitudinal measuring direction, because the observed intensity was higher than the intensity of the (1 1 1) peak. The tensile device was assembled so that the incoming synchrotron X-ray beam with a beam wave length of 0.177 nm and a beam size of 300 μm in diameter was impinged on the sample surface in reflection geometry. As shown in Fig. 1a, the Cu film stresses parallel (in the longitudinal direction) as well as perpendicular to the tensile direction (in the transverse direction) were calculated using the sin^2^ *ψ* method [28]. Therefore, a Hill model and the X-ray elastic constants (½ *S*_2_) [29] for an untextured (1 1 1) and (2 0 0) Cu reflection were applied. From these experiments, stress–strain curves averaged over the beam footprint on the sample are calculated, where the Cu film stresses obtained by the synchrotron data are plotted against the engineering strains measured with the tensile device. It should be noted that the film stresses in longitudinal and transverse directions were measured using two different samples by turning the tensile device to 90°, with one sample being measured at 0° stage rotation and a second sample measured after the stage was rotated by 90°. The stresses in the Cr interlayer and bare Cr film could not be measured with this setup in reflection geometry because with 10 nm thickness the film is too thin to receive a signal sufficient for in situ measurements.

The in situ diffraction experiments were compared to in situ straining experiments performed under an atomic force microscope (AFM) [30,31]. In situ AFM straining allows for the characterization of the surface deformation as a function of applied strain in the form of localized thinning (necking) and through thickness cracks (channel cracks) in Cu and Cu/Cr films. The crack spacing in the Cu and Cu/Cr films was determined from AFM images and the method explained in Ref. [31]. In this case, a drop in the height profile of 15% of the film thickness was defined as a through thickness crack, otherwise a neck is present. With this definition, one is able to distinguish between cracks and necks.

The post-analysis of the deformed samples was performed in a Zeiss AURIGA® CrossBeam® workstation using the focused ion beam (FIB) for milling cross-sections into the material to observe the crack propagation along the film thickness of the Cu/Cr films with the scanning electron microscope (SEM). To reduce the drift due to the electron beam interactions with the non-conductive polymer substrate, the film surfaces were sputtered with 2 nm carbon before the FIB milling. In order to study the deformation behavior of the Cr interlayer and how the Cr interlayer influences the failure of the overlying Cu film, the Cu films of the Cu/Cr samples were etched away to uncover the Cr interlayer using an electrolyte consisting of 10 ml HNO_3_, 1 ml HCl, 90 ml H_2_O and approximately 3 ml H_2_O_2_. From SEM images of the etched Cu/Cr films, one is able to measure the crack spacing of the revealed 10 nm Cr interlayer to compare it with the crack spacing of the Cu and Cu/Cr films. The crack spacing of the revealed Cr film was determined by measuring the distance between cracks along the same line profile perpendicular to the cracks using the program ImageJ [32].

The bare 10 nm Cr film was strained using a Kammrath & Weiss small scale straining device. The Cr samples were slightly larger than the Cu and Cu/Cr film systems with dimensions of 45 mm × 8 mm and a faster displacement rate of 10 μm/s was employed. Three samples were strained to 14% with SEM micrographs made of the fractured films after removal from the straining stage. The crack spacing and density at 14% were determined the same way as the buried Cr films from the Cu/Cr film systems.

### 2.2 Finite element model

In order to explore some mechanisms behind observations in the experiments, finite element (FE) studies were performed. The FE model developed for this purpose is schematically sketched in Fig. 1b. It represents a sufficiently long (in the *x*-direction) slice, cut out from the Cu/Cr specimen in the longitudinal direction (cutting plane is *x*–*z*-plane), modeled under generalized plane strain conditions, i.e., strain coupling by assuming homogeneous distribution of the transverse strains *ε_yy_*, while *ε_xy_ *= *ε_yz_* ≡ 0. These assumptions are justified for all regions of the sample, except a very narrow boundary layer at the free edges of the specimen along the *x*-direction. Furthermore, the model assumes perfect bonding between the layers. The evolution of the crack pattern up to saturation appears to be a stochastic process, in which the appearance of each individual crack in the individual Cr and Cu films is predominantly triggered by three characteristics:

(1) the longitudinal stress distribution in the film,
(2) the spatially inhomogeneous distribution of parameters in the film (thickness variations, morphology, flaws, etc.),
(3) the influence from neighboring films (e.g. cracks existing or appearing in adjacent films).

Items 1 and 3 are strongly coupled, and Item 2 makes the process a stochastic one. The formation of cracks is modeled by the introduction of cohesive zone elements [33]. For taking the stochastic character of the evolution of the crack pattern into account (neither the position nor the global strain level of initiation of the individual cracks can be predicted accurately), a large number of potential positions of cracks is introduced (around 400 out of which around 10 become active). These potential positions are modeled by cohesive zone elements, arranged in a distance of every two FE elements (see Fig. 1b) and configured with statistically varied strength parameters according to

(1) $$\sigma_{\mathrm{DI}\text{,}i\text{,}m}=\sigma_{u\text{,}m}+s_{m}n_{i\text{,}m}\text{.}$$

**Fig. 2:**
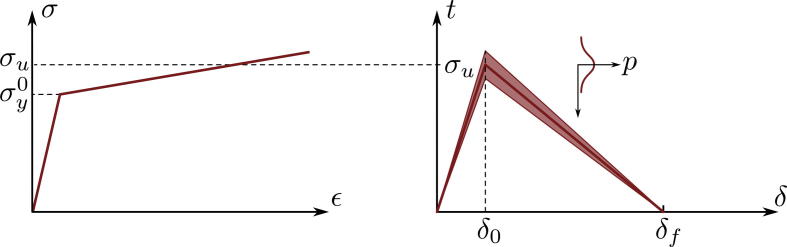
Schematic representations of the traction separation law (right) and of the corresponding stress–strain behavior of the respective layer material (left).

Here in *σ_DI,i,m_* is the damage initiation stress in the traction separation law of the *i*-th cohesive zone element (the character of which is schematically shown in Fig. 2, right) in a film made of material *m* (for copper *m *= Cu and for chromium *m *= Cr). The strength of the material up to crack initiation is principally characterized by *σ_u,m_*. The second term on the right hand side of Eq. (1) takes the unavoidable inhomogeneous distribution of the relevant parameters (thickness variations, morphology, flaws, etc.) into account. There, *s_m_* stands for the standard deviation of the statistical deviation of the damage initiation stress from its mean value *σ_u,m_* for material *m*, and *n_i,m_* represents a value out of a Gaussian standard normal distribution with mean value *μ* = 0 and variance *σ*^2^ = 1. The samples of the standard normal distribution, *n_i,m_*, were computed in Matlab [34] which employs Marsaglia’s algorithm [35] to obtain uniformly distributed pseudo-random numbers followed by the ziggurat algorithm [36] to compute the normal distribution [37]. Two different samples for the Cu and Cr films were used, and the values for *σ_u,m_*, *s_m_*, *δ*_0,_*_m_*, and *δ_f,m_* are listed in Table 1. The values for the separation at damage initiation, *δ*_0,_*_m_*, are chosen to be very small, so that the large number of cohesive zones, which are inactive, do not influence the overall stiffness. The value of the separation at failure, *δ_f,m_*, in conjunction with *σ_u,m_*, determines the amount of energy required for the crack to form. The parameters finally used were selected by parametric studies through calibration with experimental observations.

The Cu film was modeled by an elastic-plastic material with linear, isotropic hardening (as shown in the schematic stress–strain curve in Fig. 2, left). The according material parameters (Young’s modulus, Poisson’s ratio, yield limit, hardening modulus) for Cu must be selected for a nanocrystalline microstructure. Such data have become available in the last decade, most of them being for bulk material, see, e.g., the experimental work [38] and the theoretical work [39]. However, the material in very thin films, as treated here, behaves significantly different compared to bulk material. For this situation, detailed information can be gained from [40,41]. Although the properties used in the presented simulations for the 50 nm and 200 nm films, see Table 1, do not fully correspond with data from [40], the principal trends regarding differences between bulk and thin film behavior are taken into account in the models. The assumed yield strength for the 200 nm Cu film is in good agreement with the experimental data presented in [42]. Due to the fact that the extremely thin 10 nm Cr film is highly constrained, its material behavior was assumed as fully elastic up to brittle failure by crack formation. The polyimide substrate was modeled as elastic–plastic with properties as used in [19].

For allowing crack opening in the Cr layer, which is attached to the polyimide substrate, cohesive zone elements extending into the substrate were used (Fig. 1b). The properties of the cohesive zones in polyimide were chosen such that a constant ultimate traction is maintained, i.e. *δ_f,poly_* → ∞. In this way, large plastic deformations in the polyimide underneath the cracks in Cu allow crack opening in the Cr interlayer.

The FE nodes at the boundary at *x *= 0 were constrained to be immovable in the *x*-direction and left free to move in the *z*-direction. The nodes belonging to the bottom of the fine-meshed area (see Fig. 1b) were constrained to be immovable in *z*-direction. The *x*-displacements of the nodes at the right boundary of the model were prescribed, modeling the straining of the specimen in the tension test. The displacements in the *z*-direction were left free. Generalized plane strain assumptions, i.e., constant strain in the *y*-direction (perpendicular to the model plane), were used. The whole specimen is assumed as stress free at the start of the simulation of the tensile test.

## 3 Results

### 3.1 Experiments

**Fig. 3:**
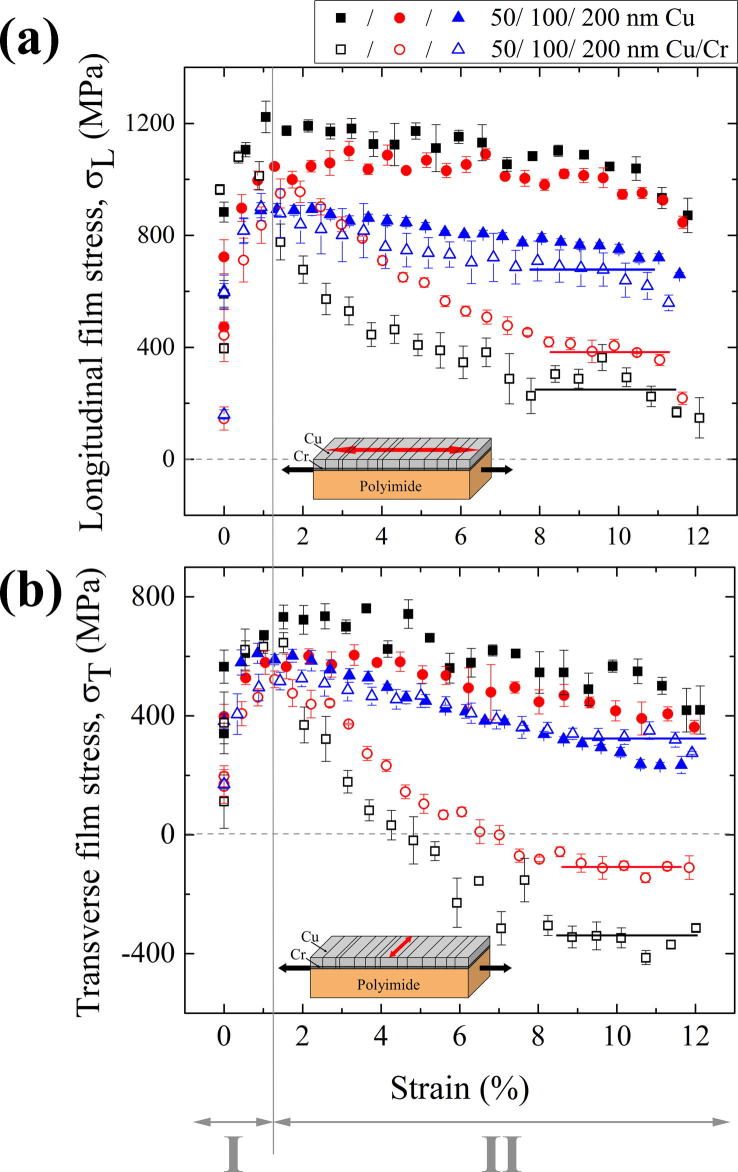
(a) Longitudinal film stresses, *σ_L_*, and (b) the transverse film stresses, *σ_T_*, in Cu films and in Cu layers of the Cu/Cr films with 50, 100 and 200 nm Cu layer thicknesses. The film stresses measured with the sin^2^ *ψ* method are plotted against the engineering strain, *ε*, determined by the tensile device. The results for Cu films are marked with full symbols and for the Cu/Cr films with open symbols. The first points of the curves correspond to the residual stresses measured before the tensile test. The inset diagrams show the tensile direction (black arrows) and the measuring direction of the film stresses (red arrow). The standard deviation is used to calculate the error bars. In order to guide the eye, the solid lines indicate the plateaus of the crack saturation region in the Cu/Cr films. Data from (b) are referred to [14].

The film stresses of all Cu (full symbols) and Cu/Cr films (open symbols), measured in longitudinal direction as well as in transverse direction, are shown in Fig. 3, where the first point of each curve at zero strain correlates to the residual stresses, determined before the tensile test. Fig. 3a shows the film stresses resulting from measurements in the longitudinal direction and Fig. 3b in the transverse direction, as indicated by red arrows in the inset diagrams. For all film systems, the stresses increase with increasing strain up to the maximum peak stresses (*σ_L,max_* and *σ_T,max_*) which are listed with the corresponding strains (*ε_L,max_* and *ε_T,max_*) for each direction and sample set in Table A.1 in the Appendix A. After having reached the peak stresses (Region I), the film stresses start to decrease (Region II). In the case of the Cu films, the stresses slightly decrease, and in the case of the Cu/Cr films, the stresses rapidly decrease for strains up to about 7%, until the maximum strain of 12% is reached.

The stresses of all films in the longitudinal direction (Fig. 3a) lie in the tensile regime, and the pure Cu films show higher stresses for all film thicknesses compared to the Cu/Cr films. A similar trend can be observed in the transverse direction (Fig. 3b), where the measured stresses of the 50 and 100 nm Cu/Cr films even drop into the compressive range. It could be shown that the transverse compressive stress of the 50 nm Cu/Cr film is high enough to induce delamination of the film system from the substrate in the form of buckles [14]. For the 200 nm Cu and Cu/Cr films nearly the same film stresses are measured (blue open and full triangles). The agreement is observed in the longitudinal as well as in the transverse straining direction. This is, however, not the case for the other film thicknesses. It should be noticed that the film stresses of the bare 50 nm Cu films remain higher than that of the 200 nm Cu films. In contrast, the film stresses of the 50 nm Cu/Cr film continuously drop after a few per cent of deformation to much lower film stresses than the 200 nm Cu/Cr film.

**Fig. 4:**
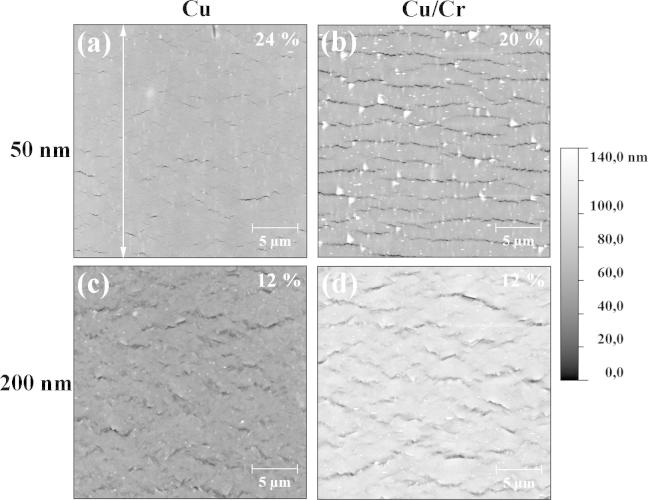
Post-analyzed AFM images of the (a) 50 nm and (c) the 200 nm Cu film, and, (b) of the 50 nm and (d) 200 nm thick Cu layer of the Cu/Cr films at different strains. The straining direction is indicated by the double sided arrow.

**Fig. 5:**
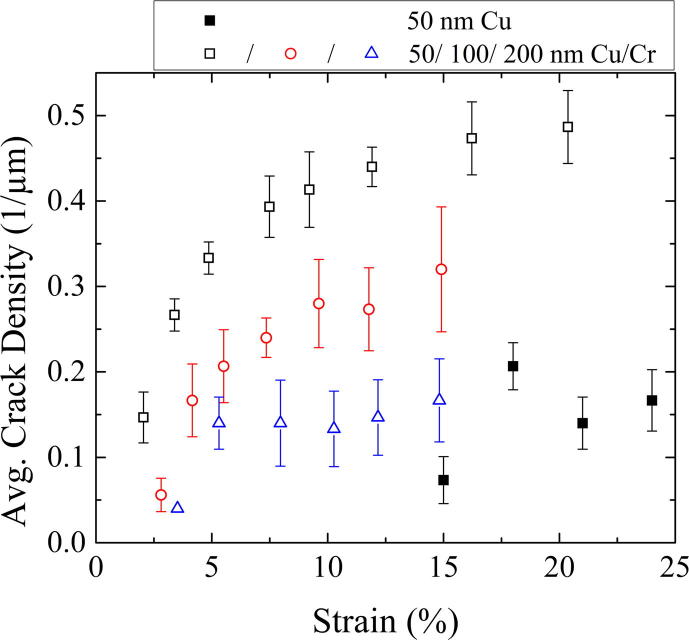
The averaged crack densities against the engineering strain of the bare 50 nm thick Cu film (full symbols) and the 50, 100 and 200 nm thick Cu films of the Cu/Cr films (open symbols).

Fig. 4 shows post-strained AFM images of the surface topography of the deformed 50 and 200 nm Cu and Cu/Cr films at different strains. The 50 nm Cu film exhibits a smaller amount of short cracks at a higher strain in comparison to the 50 nm Cu/Cr film with a higher amount of longer straight cracks at a lower strain. Both 200 nm films show deformation in the form of necking, but with some cracks in the Cu/Cr film, which had initially started as regions of necking. However, the necks in the 200 nm Cu/Cr film appear to be straighter and longer than the short zig–zag shaped neck contours of the 200 nm Cu film. It should be mentioned that, within the considered range of straining, cracks in Cu films formed only in the 50 nm thick film. All of the deformation observed in the 200 nm Cu film was only in the form of necking, and no through thickness cracks were observed. The experimentally determined development of the average crack density from the in situ AFM experiments is shown Fig. 5 for the Cu/Cr films. One can clearly see that for all Cu film thicknesses the saturation of the crack formation is achieved at an engineering strain of about 12–15% having a substantial influence on the development of the crack density: the thinner the Cu film, the higher the crack density. Also shown in Fig. 5 is the crack density evolution of the 50 nm Cu film without the Cr interlayer. This was the only bare Cu film in which through thickness cracks were observed. Of further interest is that through thickness cracking did not occur until 15% strain, when saturation of the Cu/Cr film was reached. From these four films, the crack spacing of the bare 50 nm Cu film is 5.1 ± 4.3 μm (at 15% strain), and for the Cu/Cr samples, the crack spacing is 2.0 ± 0.7 μm for the 50 nm (at 16% strain), 2.9 ± 1.4 μm for the 100 nm and 5.5 ± 3.6 μm for the 200 nm thick Cu layer (both at 15% strain), measured from height profiles of AFM images using the method outlined in [31].

**Fig. 6:**
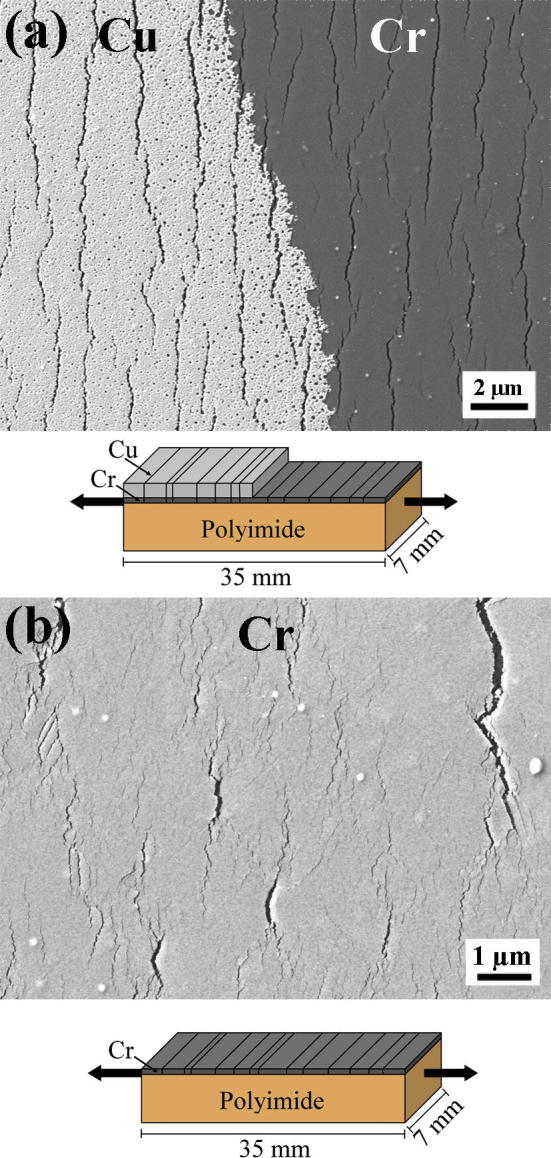
SEM images of the etched sample surfaces strained to 12% with the corresponding sketches of the etched area. (a) Cu/Cr film with a 50 nm thick Cu layer (both layers of the film system the Cu layer and the underlying Cr interlayer are visible) and (b) Cu/Cr film with a 200 nm thick Cu layer (only the Cr interlayer is visible).

**Fig. 7:**
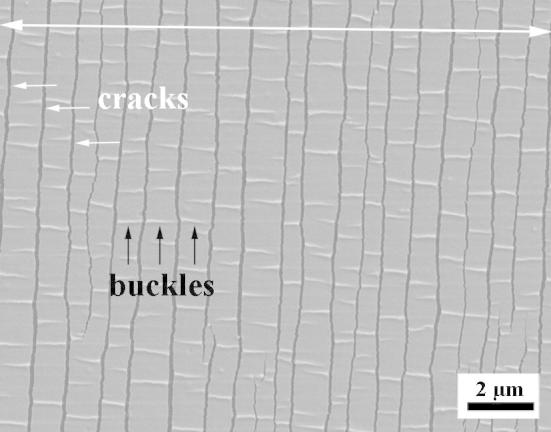
SEM image of a 10 nm thick Cr film strained to 14%. Cracks form perpendicular and buckles parallel to the straining direction, marked with a double sided arrow.

In order to link the deformation of the Cu surface with the crack network of the underlying Cr interlayer, the deformed Cu/Cr films were etched. In Fig. 6, the SEM images of the etched 50 and 200 nm thick Cu/Cr films strained to 12% are shown with the corresponding diagrams of the etched areas. From the SEM micrographs, it can be concluded that the fracture pattern of the Cr interlayer changes with increasing Cu film thickness. In the case of the 50 nm Cu film, the Cr interlayer shows the same crack morphology as the overlying Cu film, while the Cr interlayer of the 200 nm Cu film shows some larger cracks in between a fine crack network. The crack spacing is 1.48 ± 0.19 μm for the Cr interlayer of the 50 nm Cu layer, 1.39 ± 0.17 μm for the Cr interlayer of the 100 nm thick Cu layer, and 0.51 ± 0.11 μm for the Cr interlayer of the 200 nm thick Cu layer of the Cu/Cr films, all strained to 12%. The values of the crack spacing in the Cr interlayer decrease with increasing Cu layer thickness and are similar or slightly greater than that for the bare 10 nm Cr film on polyimide, which was measured to be 0.88 ± 0.2 μm (at 14% strain). An SEM image of the bare 10 nm Cr film strained 14% shows that Cr forms a high amount of straight channel cracks leading to the small crack spacing. The film segments between neighboring channel cracks show delaminations in the form of buckles perpendicular to the cracks (see Fig. 7). The mechanism of this kind of structural instability is discussed in detail in Refs. [18,19].

**Fig. 8:**
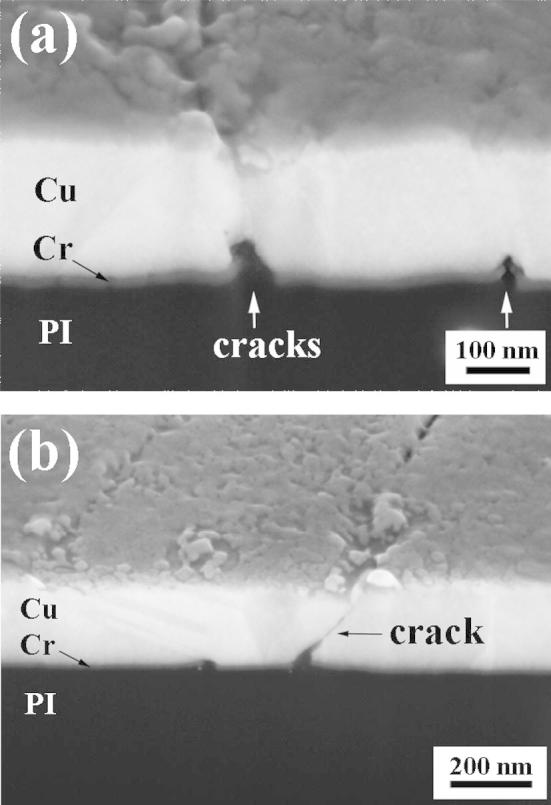
FIB cross section images of a Cu/Cr film with a 200 nm thick Cu layer, strained to 18% in a SEM. (a) Cracks in the Cr interlayer are visible and marked with white arrows; the overlying Cu layer is intact without cracking. (b) A crack propagated from the Cr interlayer through the Cu layer.

By FIB cross sectioning, one is able to provide information about the deformation not only on the film surface but also through the film thickness. Fig. 8 shows SEM images of the FIB cross-sectioned Cu/Cr film with a 200 nm thick Cu layer. It could be observed that the Cr interlayer forms a high number of cracks. These cracks are either blunted at their entrance into the Cu layer (Fig. 8a) or can eventually propagate through the Cu film (Fig. 8b). These observations are further evidence that the Cr interlayer greatly influences the mechanical behavior of the overlying ductile film.

### 3.2 Simulations

Applying the simple shear lag model from Ref. [43,44] on the experimental results fails to explain the crack formation behavior of Cu/Cr films, for which two interacting interfaces, the Cr-substrate interface and the Cr–Cu interface, must be taken into account. Therefore, finite element simulations were performed: (i) to better understand the differences between the deformation behavior of pure Cu films and Cu/Cr films at higher strains, and (ii) to provide comparisons with the experimental findings.

**Fig. 9:**
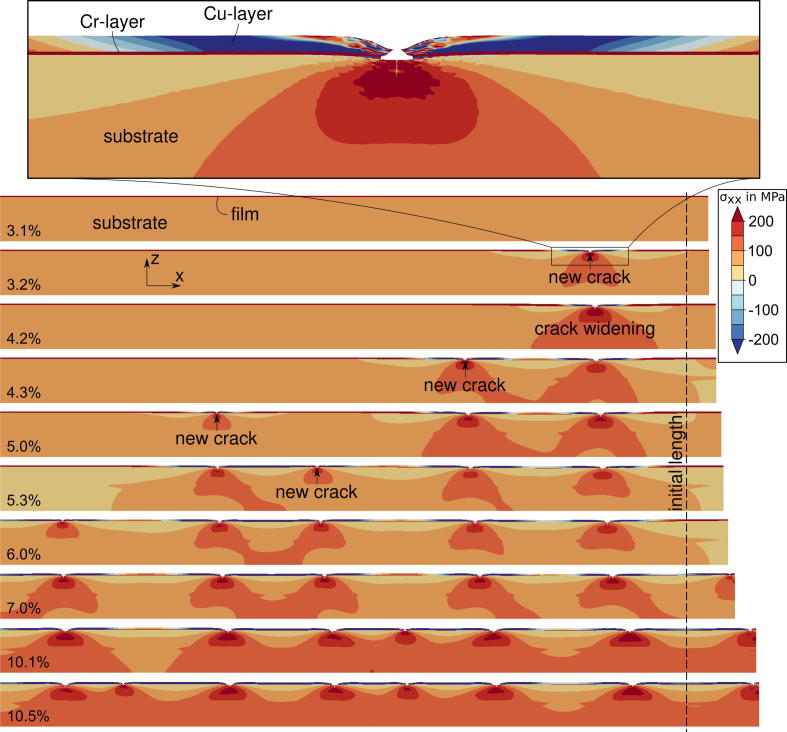
Evolution of the crack pattern in Cu/Cr films by straining of the specimen. Simulation results obtained for the model with a 50 nm thick Cu layer. Colors show the highly inhomogeneous distribution of longitudinal stresses, *σ_xx_* (MPa). The inset shows an enlarged area of a through thickness crack at 3.2% strain. (For interpretation of the references to colour in this figure legend, the reader is referred to the web version of this article.)

**Fig. 10:**
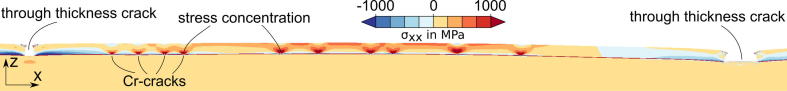
Locations of cracks in Cr and Cu, respectively, for the Cu/Cr film with a 200 nm Cu layer. Colors show the longitudinal stresses *σ_xx_* (MPa). (For interpretation of the references to colour in this figure legend, the reader is referred to the web version of this article.)

FE simulations performed with the model described in Section 3 have provided relevant information for interpreting the experimental observations, in particular with respect to the evolution of the crack pattern in the Cu/Cr film. In Fig. 9, a sequence of simulations for a 50 nm Cu/Cr sample deformed at increasing engineering strains up to 10.5% is shown. The first crack appears in the Cr interlayer at the position of the weakest cohesive zone in Cr. This crack induces a stress concentration in the Cu film, which leads to cracking at the same location (in the example shown in Fig. 9 at 3.2% global strain). For the 50 nm as well as for the 100 nm thick Cu films, cracks in Cu appear in the same positions at which cracks in the Cr film have already formed. This occurrence of Cr and Cu cracks was experimentally observed (see Fig. 6a). However, the same cracking behavior is not observed in the Cu/Cr film with a 200 nm thick Cu film, where the crack pattern in the Cu film shows crack distances larger than in the underlying Cr film, as can be seen in Fig. 10.

**Fig. 11:**
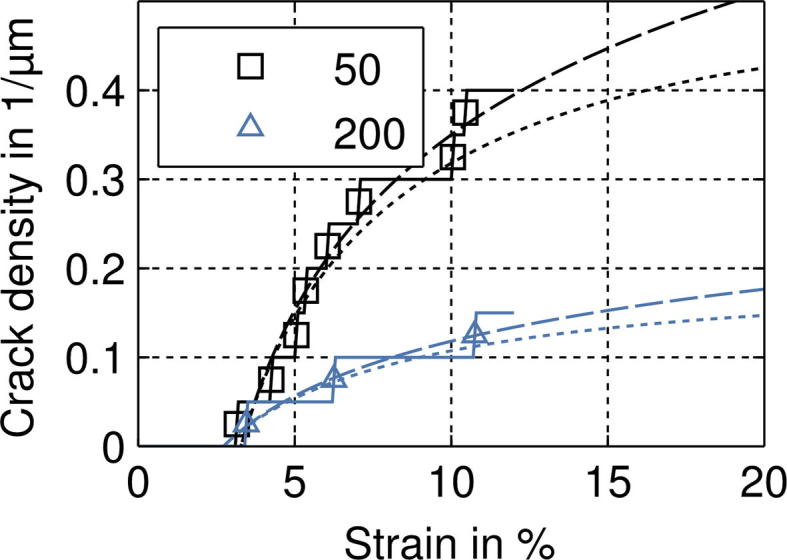
Simulated crack density for different Cu film thicknesses. The dotted lines show fit curves of computed crack densities (reference length according to the current, i.e. strained configuration). The experimental data can be seen in Fig. 5 to demonstrate how well the model and experiment coincide.

Fig. 11 shows the development of the crack density for the 50 nm and the 200 nm thick Cu layer of the modeled Cu/Cr systems. The crack density is lower for thicker Cu films than for thinner films, and shows the same behavior as observed in the experiments (Fig. 5). Only a few cracks in the Cr film, which might act as crack initiators in the 200 nm Cu film, lead to fully formed Cu cracks (see Fig. 10).

**Fig. 12:**
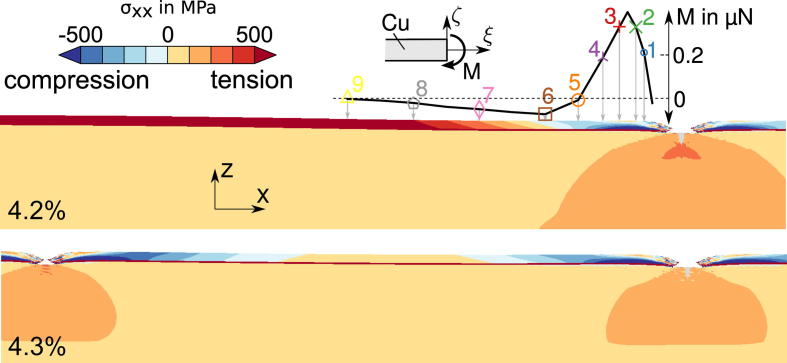
Computed longitudinal stress distributions (*σ_xx_* in MPa) for the Cu/Cr system with a 50 nm thick Cu layer. Stress redistribution near the crack front due to generation of a free surface (top). Bending moment per unit thickness in the Cu layer with respect to the center (insert graph); stress re-distribution due to springback after formation of a neighboring crack (bottom).

**Fig. 13:**
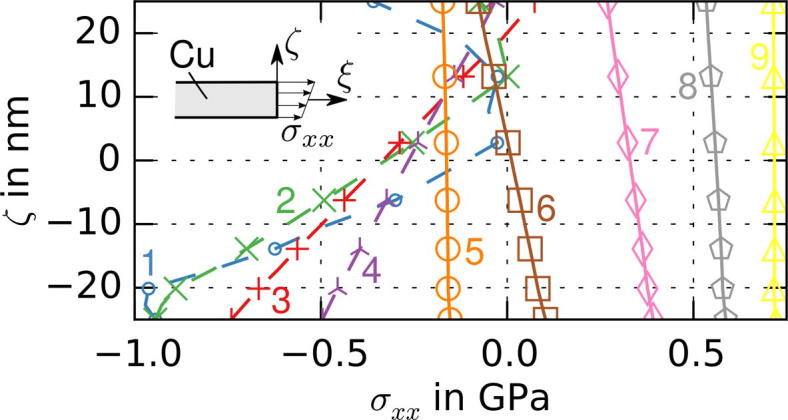
Distribution of longitudinal stress, *σ_xx_*, over the thickness of the Cu layer for different cross sections of the 50 nm thick Cu film at 4.2% global strain. The positions of the sections are shown in Fig. 12.

The simulations also show that the stress distribution in the cracked film is highly inhomogeneous (see Fig. 12). The development of the stress distribution in the Cu film can be described as follows: Before any crack has formed, a homogeneous stress state exists in the model. When the films crack, a free surface is formed (requiring *σ_xx_* = *σ_xy_* = *σ_xz_* = 0 along the crack face) leading to unloading of the films in the longitudinal direction (the spring back). The distribution of the longitudinal stress over the thickness of the Cu film is shown in detail in Fig. 13 for several cross sections whose position is indicated in Fig. 12. The bending moment in the Cu film can be computed from the stress distribution (see Fig. 12). Two areas can be distinguished: High compressive stresses arise in cross sections close to the crack (1–4), because the plastically deformed Cu film, which now has a longer stress-free length, relaxes to the same length as the elastic Cr film. This also leads to a positive bending moment responsible for a slight downward curvature of the film. Distances further from the crack (Sections 6–9) shear stresses at the polyimide–Cr interface (the shear lag), transferred to the Cu film via the Cr interlayer, cause the gradual restoration of the homogenous stress state. Because the shear stresses act on the lower interface of the film system, a negative bending moment is introduced (see [19]). The effect of the bending moment is only significant in areas with low average tensile stress (Sections 2–6) and diminishes as the distance from the crack increases. As the next crack arises a similar stress re-distribution commences (see Fig. 12, below).

**Fig. 14:**
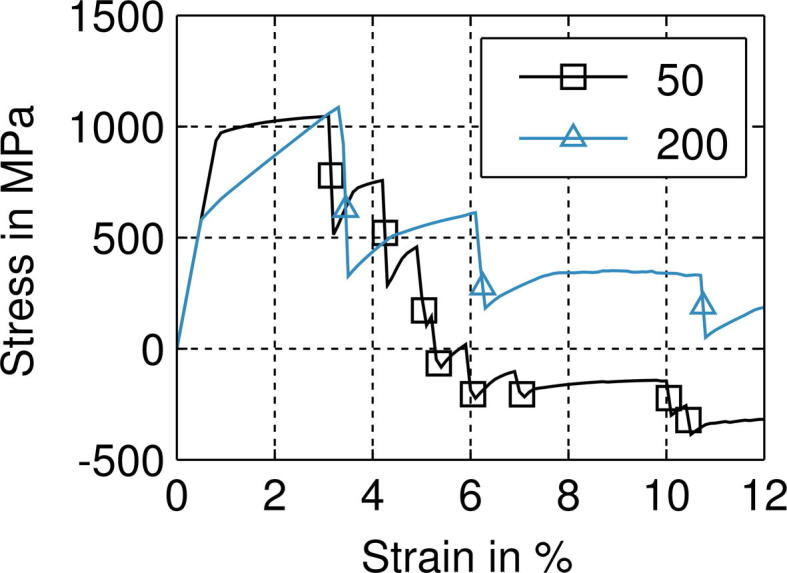
Development of the computed longitudinal film stress in 50 and 200 nm Cu layers of Cu/Cr films. For comparison reasons, the shown stress values (in MPa) represent element size weighted means of the computed inhomogeneous longitudinal stress distribution–compare with Fig. 3.

Because of the inhomogeneity of the stress distribution, the volume weighted average of the stress values over all Cu elements in the model was computed and is displayed in Fig. 14 as a function of the global strain. Thicker Cu films show a higher averaged longitudinal stress as compared to the values for thinner films. Thus, the principle trends regarding the influence of film thickness observed in the experiments was also represented by the model. The zig–zag character of the diagram in Fig. 14 comes from the fact that the simulation model represents only a very small detail from the much longer specimen. In the model, every appearance of a crack leads to a sudden drop of the averaged longitudinal stress, while in much longer specimens used in the experiment this effect is smeared out, resulting in a rather smooth curve. It should be mentioned that, since large strains are involved, the same reference length (deformed or initial configuration) must be used when measured crack densities are compared with calculated ones.

## 4 Discussion

The typical cracking behavior of brittle-like metal films on polymer substrates was observed in all studied Cu and Cu/Cr films, but is more pronounced in the Cu/Cr films due to the higher density of cracks that formed in comparison to the Cu films, which shows substantial strain localization via neck formation (see Figs. 4 and 5). Other ductile film systems have also shown brittle-like behavior in the presence of a brittle interlayer, for example Cu with a Ti interlayer [7], Cu with a Ta interlayer [10], and Au with a Cr interlayer [45]. From the experimental results (Fig. 3a), one can see that at higher strains the deformation behavior of the Cu films differs substantially in character from that of the Cu/Cr films. In the longitudinal direction, the Cu/Cr film stresses decrease more toward compressive stresses with decreasing film thickness, while the bare Cu films show the opposite behavior, namely the thicker films show lower longitudinal film stresses than the thinner films. For the bare Cu films, it is assumed that the film thickness and the as-deposited grain size dominate the behavior in regions I and II. This is the reason behind the 50 nm film having the largest peak stress and saturation stress compared to the 200 nm Cu film, since this film has the smallest grain size. It should be mentioned that for pure Cu and Cu/Cr films the Cu grain sizes are very similar. This leads to stress values which increase equally for the same Cu film thicknesses (i.e. 50, 100 or 200 nm of Cu) with increasing strain (Region I) up to the point where the brittle Cr layer causes early necking or cracking in the attached Cu films. After the films deform via necking or cracking, the measured film stresses are averaged values for the inhomogeneous stress field and are, thus, not representative for the strength of the Cu layer.

In the case of the Cu/Cr film system, the deformation behavior is influenced by two different mechanisms depending on the strain. At low strains as indicated by Region I, the deformation behavior is influenced by the film thickness and grain size similar to the bare Cu films, but at higher strains (Region II), especially in the saturation regime where it is known that the Cu/Cr films are cracked, the deformation behavior is most likely dominated by the Cr interlayer (see Fig. 3).

In the transverse direction, the film stresses start in the tensile regime and initially increase with increasing strain like the stresses in the longitudinal direction (see Fig. 3b). This behavior can be explained by the 2D stress analysis by Frank et al. [9] that was applied to Ta films on polyimide strained in situ at a synchrotron. The transverse coating stresses increase due to a slight mismatch of the different Poisson’s ratios of the film and the substrate until the film starts to crack. Then a stress relaxation occurs in the longitudinal direction, and simultaneously a reduction in the transverse stress, which can even become compressive. The transverse stresses predicted by the FE model were considerably lower than the measured values due to the assumed Poisson’s ratios. However, this does not impact the significance of the results in the longitudinal direction.

The stress measured by X-ray diffraction can be regarded as an average value over the beam area. For the bare Cu films, which do not show cracks for strains below 10%, the measured average stress will correspond well to the ultimate values in the sample. However, in the Cu/Cr samples the brittle Cr layer cracks at low strains, which introduces stress concentrations: the ultimate stress in the Cu layer is highly localized around cracks in the Cr layer. These stress concentrations lead to cracking of the Cu layer, which causes partial unloading and, thus, a decrease in the average stress. This explains why the measured stresses in the Cu/Cr system are lower than for bare Cu films.

The FE simulations help to shed light on the fact that for the Cu/Cr samples the thicker Cu films exhibit higher values of averaged longitudinal stresses in the crack saturation range than thinner films. When a crack is formed in the Cu film, a redistribution of the stress state due to a local unloading occurs reducing the longitudinal tensile stress in the Cu film which leads to a new state of equilibrium. The purely elastic Cr film, which is perfectly bonded to the Cu film, releases most of its elastic strain energy and compresses the plastically elongated Cu film between neighboring Cu cracks. Certainly, also the polyimide substrate contributes to this stress redistribution effect, which is quite the same in all configurations, so it is no longer discussed in the following context.

The Cr film has the same layer thickness (10 nm) in all considered configurations and shows an overall strain-dependent crack pattern, which is very similar for all considered Cu film thicknesses. Thus, the elastic strain energy, available from stress redistribution, would be also similar for all Cu film thicknesses. However, while in the case of thin Cu films the crack opening in the Cu and Cr films coincide, this is not the case for thicker Cu films, where many Cr cracks underneath the Cu film are hindered to open substantially (see Figs. 8a and 10), and less elastic strain energy is released from the Cr film. In order to get the same effect of stress redistribution for thicker Cu films as it occurs for thinner films, even more elastic strain energy released from the Cr film would be required. However, this is not the case and, hence, the reduction of the averaged longitudinal stress in thicker Cu films is smaller. Consequently, the averaged longitudinal stress according to the saturated crack density is higher in the thicker Cu films.

Furthermore, the regions of high values of local longitudinal compressive stresses in Cu films are close to the edges of the individual Cu strips between neighboring channel cracks (see the blue zones in Fig. 12). Due to higher crack densities in thinner Cu films, these regions of local longitudinal compressive stresses in Cu occupy more Cu volume than in thicker Cu films, leading to larger contributions of compression in the volume averaged longitudinal stress value. The higher crack densities in thinner films are a result of the higher amount of energy available for fracture and the longer shear lag of thicker films.

The finite element simulations also revealed a highly complex stress state with large variations of values over only a few nanometers in the cracked copper film, resulting as an effect of straining above the yield limit, subsequent cracking and stress redistribution, and further straining. Therefore a volume weighed average of the stresses was computed in order to allow comparison with experimentally measured stress values. This way of averaging is most likely only a rough estimate for that what the sin^2^ *ψ* method with the comparably large beam size (a diameter of 300 μm) is doing in terms of stress averaging. Nevertheless, the principal trends regarding the influence of film thickness observed in the experiments were also found in the averaged stress results of the simulations.

## 5 Conclusions

In this study, in situ tensile tests in the synchrotron as well as under an AFM were performed on various thicknesses of Cu films, with and without a Cr interlayer, to investigate the deformation behavior such as the crack morphology and the stress state during straining with the influence of the brittle interlayer of particular interest. The experiments have shown that the Cr interlayer dominates the deformation behavior. At low strains, all film stresses of the bare Cu and the Cu/Cr films are influenced by the film thickness and the grain size, the thinner the films the higher the maximum peak stresses (see Fig. 3, Region I). At higher strains (in the crack saturation range), the two film systems start to behave differently. In Region II, the Cu/Cr films are influenced not only by the film thickness and grain size but also by the brittle Cr interlayer. Bare Cu films still follow the “smaller is stronger” trend where the thinnest film with the smallest grain size exhibits the highest film stresses. In contrast, the Cu/Cr films show the opposite trend where the thinnest films have the smallest film stresses in the crack saturation range. In this case, the brittle Cr interlayer forms cracks which induce a stress concentration in the overlying Cu film, where the cracks can either be blunted by the Cu film or can propagate into the Cu film. It can be concluded that usually all adhesion promoting interlayers which are brittle, such as Cr, Ti or Ta, will initiate failure in the attached film upon exposure to mechanical loading [7,10,45].

The FE simulations qualitatively explain the behavior of the Cu/Cr films. The formation of cracks is responsible for the reduction of the averaged longitudinal stress, and the plasticity of the Cu layer plays an important role in this process. The higher crack density in the Cu/Cr films, thus, explains the larger drop in longitudinal stress as compared to Cu films. Cracks are triggered by the brittle fracture of the Cr layer, which introduces a stress singularity in the Cu layer, leading to a through thickness crack. If a crack penetrates the complete film thickness, the stresses on the edges of the crack redistribute to a highly inhomogeneous stress state in the Cu islands between two cracks and a shear lag behavior is formed.

## Figures and Tables

**Table A.1:** Summary of the measured stresses from the in situ synchrotron experiments. *L* represents the data measured in the longitudinal direction and *T* represents the data measured in the transverse direction. In the table, *h*_Cu_ is the film thickness of the Cu films.

	*h*_Cu_ (nm)	*σ*_res_ (Mpa)	*σ_L,_*_max_ (Mpa)	*ε_L,_*_max_ (%)	*σ_T,_*_max_ (Mpa)	*ε_T,_*_max_ (%)	*σ_L,_*_10%_ (Mpa)	*σ_T,_*_10%_ (Mpa)
Cu	50	591 ± 48	1222 ± 56	1.06	732 ± 40	1.51	1038 ± 42	567 ± 20
	100	473 ± 17	1046 ± 3	1.28	579 ± 40	1.05	946 ± 17	417 ± 34
	200	602 ± 25	893 ± 21	1.34	610 ± 34	0.86	751 ± 17	277 ± 16

Cu/Cr	50	396 ± 17	1079 ± 21	0.37	646 ± 34	1.51	293 ± 33	−347 ± 34
	100	145 ± 42	955 ± 38	1.93	522 ± 28	1.29	381 ± 4	−104 ± 15
	200	160 ± 16	901 ± 49	0.94	525 ± 28	1.98	639 ± 61	328 ± 25

**Table 1:** Data of the material and cohesive zone parameters used in the FE simulations.

		Chromium	Copper 200 nm	Copper 50 nm
*E*	Young’s modulus (GPa)	279.0	120.0	120.0
*v*	Poisson’s ratio	0.21	0.30	0.30
$\sigma_{Y}^{0}$	Yield limit (GPa)		0.6	1.0
*E_t_*	Hardening modulus (GPa)		20.0	1.0
$\sigma_{u\text{,}m}$	Damage initiation (GPa)	12.0	2.6	1.13
$s_{m}$	Standard deviation (GPa)	1.2	0.26	0.02
*δ*_0_	Separation at damage init. (nm)	0.00543	0.00113	0.0026
*δ_f_*	Ultimate separation (nm)	0.3	10.0	0.3

## Appendix A

Table A.1 contains the film stress data measured in situ using synchrotron diffraction for all of the Cu and Cu/Cr samples strained in the longitudinal and transverse directions (subscripts *L* and *T*, respectively). The maximum peak stresses (*σ_L,max_* and *σ_T,max_*) are listed with the corresponding strains (*ε_L,max_* and *ε_T,max_*) along with the residual stresses (*σ_res_*) and the film stresses at 10% strain (*σ_L,_*_10%_ and *σ_T,_*_10%_).
